# Fabrication of Bioactive, 3D-Printed, Porous, Yttria-Stabilized Zirconia via Mg/Zn-Incorporated Modified Simulated Body Fluid Pretreatment

**DOI:** 10.3390/ijms262210950

**Published:** 2025-11-12

**Authors:** Yuwei Wu, Shigeomi Takai, Takeshi Yabutsuka

**Affiliations:** Graduate School of Energy Science, Kyoto University, Kyoto 606-8501, Japan; wu.yuwei.37w@st.kyoto-u.ac.jp (Y.W.); stakai@energy.kyoto-u.ac.jp (S.T.)

**Keywords:** yttria-stabilized zirconia, modified simulated body fluid, magnesium, zinc, bioactivity, hydroxyapatite

## Abstract

Yttria-stabilized zirconia (YSZ) has attracted attention as a ceramic implant material owing to its excellent mechanical strength, biocompatibility, and aesthetic properties. However, YSZ is bioinert and lacks the ability to directly bond with bone. This study aims to enhance the bioactivity of 3D-printed porous YSZ through modified simulated body fluid (m-SBF) pretreatments. The porous YSZ substrates fabricated by fused deposition modeling were first etched with hydrofluoric acid (HF) to increase the surface roughness, followed by immersion in CO_3_^2−^, Mg^2+^, and/or Zn^2+^ ion-incorporated m-SBFs. Among the tested solutions, the apatite coating formed in Mg^2+^- and Zn^2+^-containing m-SBF within one day, exhibiting uniform precipitation and a reduced tetragonal-to-monoclinic (t→m) transition. The incorporated Mg^2+^ and Zn^2+^ ions were successfully detected on the apatite coating, with Mg/Ca and Zn/Ca ratios of approximately 4.82% and 3.33%, respectively. Mg^2^⁺ is known to stimulate osteogenesis, while Zn^2^⁺ exhibits antibacterial activity. Furthermore, compared with standard SBF under high-temperature and high-pH conditions, the m-SBF induced markedly less t→m phase transition on YSZ substrates, suggesting that m-SBF, as a biomimetic medium for imparting bioactivity, provides a more suitable environment for YSZ substrates. This study demonstrates that HF surface treatment combined with Mg^2+^- and Zn^2+^-containing m-SBF pretreatment effectively imparts bioactivity to 3D-printed YSZ, offering a promising approach for next-generation osteoconductive ceramic implants.

## 1. Introduction

Zirconia (ZrO_2_) has been extensively applied in the biomaterial field, particularly in orthopedics and dentistry, due to its excellent biocompatibility, chemical stability, and high mechanical strength. Unlike metallic biomaterials, ZrO_2_ offers superior aesthetic properties owing to its tooth-like color, lower plaque accumulation [1], and freedom from artifacts during magnetic resonance imaging diagnosis. These properties make ZrO_2_ an alternative or a complement to metallic implant materials, and there is growing clinical interest and continuing progress in biomaterials research to further enhance its performance.

Despite these advantages, pure ZrO_2_ is susceptible to low-temperature degradation, which is caused by a spontaneous tetragonal-to-monoclinic (t→m) phase transition under humid conditions. This phenomenon promotes surface microcracking and strength degradation, ultimately limiting its long-term clinical reliability [2].

To overcome these limitations, yttria-stabilized zirconia (YSZ) was developed. The incorporation of Y_2_O_3_ effectively maintains the tetragonal phase at room and physiological temperature, thereby suppressing premature t→m transition. This stabilization mechanism not only prevents hydrothermal aging but also enables transition toughening at crack tips, significantly enhancing fracture toughness and crack resistance [3].

Building on this stabilization mechanism, YSZ demonstrates superior long-term durability under clinical conditions. Importantly, these mechanical improvements are achieved without compromising the inherent biological advantages of ZrO_2_. YSZ retains its excellent biocompatibility, low bacterial adhesion, and favorable soft-tissue responses, and it also provides improved longevity and structural reliability in vivo [4]. Consequently, YSZ has become the predominant ZrO_2_-based ceramic for orthopedic and dental implants.

To further enhance its clinical performance, recent studies have investigated the role of porous structures in improving biological integration. Marques et al. [5] reviewed current technologies for bioceramic scaffolds, emphasizing the need to balance porosity, pore size, and mechanical stability, and highlighting additive manufacturing as a promising tool for tailored designs. Similarly, Rodrigues et al. [6] summarized the biological and mechanical performance of porous ZrO_2_, finding that high porosity with interconnected macro- and micropores promotes vascularization and bone ingrowth while maintaining sufficient strength for repair. These findings indicate that high porosity can partially compensate for the limited biological response of ZrO_2_ by promoting tissue ingrowth.

Although ZrO_2_ is biocompatible and possesses excellent mechanical properties, it lacks intrinsic bioactivity, which limits its ability to directly bind to bone. Apatite coatings, particularly hydroxyapatite, have been widely used to impart the apatite-forming ability to bioinert substrates. The apatite layer promotes osteoconduction, improves initial bone attachment, and facilitates faster integration of the implant into host bone tissue [7]. This apatite-forming ability can be evaluated in simulated body fluid (SBF), where the in vitro apatite formation strongly correlates with the in vivo bone-bonding ability of implants, a property generally referred to as bioactivity [8]. When the temperature and pH of SBF are enhanced, nanoscale particles in an amorphous phase are formed. Yao et al. termed these particles apatite nuclei, which exhibit a high apatite-forming ability and can act as precursors to induce apatite crystallization [9].

The formation of apatite on hydroxyapatite surfaces has been explained by an electrostatic adsorption mechanism, as reported by Kim et al. [10]. Therefore, surface treatments that enhance the ionic adsorption capability are expected to improve the apatite-forming ability. Acid treatment is one such effective approach. In our previous work on Zr-50Ti alloys [11], sulfuric acid treatment enhanced the precipitation of calcium phosphate in modified simulated body fluid (m-SBF). In addition, this process increases surface roughness, thereby providing more nucleation sites for apatite formation.

Moreover, although SBF reproduces an ion composition similar to that of human plasma, it contains ions that do not directly contribute to apatite precipitation. To better control nucleation, the SBF composition can be modified by removing non-essential ions and introducing selected biofunctional ions. This m-SBF approach enables the design of solution environments tailored to promote both apatite formation and specific biological functionalities. For instance, in this study, Mg^2+^ and Zn^2+^ ions were incorporated into m-SBF to explore their effects on apatite formation. It has been widely reported that Mg^2+^ can stabilize amorphous calcium phosphate, retard its crystallization into apatite [12], and stimulate osteogenesis in human osteoblasts [13]. In our previous studies on coating systems based on Zr-50Ti alloys, this retardation refined the grain size and inhibited local overgrowth, resulting in a more uniform coating [14]. Incorporation of Zn^2+^ has also been reported to provide both structural and biological benefits. Ren et al. [15] found that Zn^2+^ substitution for Ca^2+^ in the hydroxyapatite lattice reduced the crystallinity and crystal size, producing defect-rich structures. Ortiz et al. [16] showed that Zn-containing hydroxyapatite coatings on titanium promoted osteoblast adhesion and proliferation while changing the crystal morphology. Additionally, Predoi et al. [17] demonstrated that Zn-doped hydroxyapatite exhibited concentration-dependent antibacterial activity against *E. coli* and *S. aureus*, whereas pure hydroxyapatite remained inactive. These studies indicate that Zn^2+^ acts as a multifunctional dopant, enhancing osteogenesis and providing antimicrobial effects.

This study aims to develop a biomimetic method to form an apatite coating on YSZ by combining hydrofluoric acid (HF) etching and Mg^2+^- and Zn^2+^-containing m-SBF immersion. This approach revealed that, compared with standard SBF, m-SBF not only enables Zn^2+^ incorporation but also significantly reduces the t→m transition, thereby mitigating low-temperature degradation. Porous YSZ substrates fabricated by fused deposition modeling 3D printing were treated with HF. The acid-treated substrates were then immersed in seven types of modified simulated body fluids (m-SBFs) (pH 8.0, 36.5 °C) and heated to 70 °C, namely, high-pH SBF for apatite nucleation (AN-SBF); Zn-SBF (Zn^2+^-containing SBF); CaP solution (containing Ca^2+^ and PO_4_^3−^); Mg-CaP solution (containing Mg^2+^, Ca^2+^, and PO_4_^3−^); Zn-Mg-CaP solution (containing Zn^2+^, Mg^2+^, Ca^2+^, and PO_4_^3−^); 2Zn-Mg-CaP solution (containing double Zn^2+^, plus Mg^2+^, Ca^2+^, and PO_4_^3−^); and C-Mg-CaP solution (containing CO_3_^2−^, Mg^2+^, Ca^2+^, and PO_4_^3−^). Through this process, the apatite-forming ability was imparted to YSZ substrates. Following the m-SBF treatment, the samples were soaked in standard SBF (pH 7.4, 36.5 °C) for 1, 3, and 7 days to evaluate their apatite-forming abilities. This procedure was employed to assess the bioactivity of m-SBF-treated YSZ substrates in accordance with ISO 23317 [18].

## 2. Results

### 2.1. Surface Morphology Before and After HF Treatment

**Figure 1:**
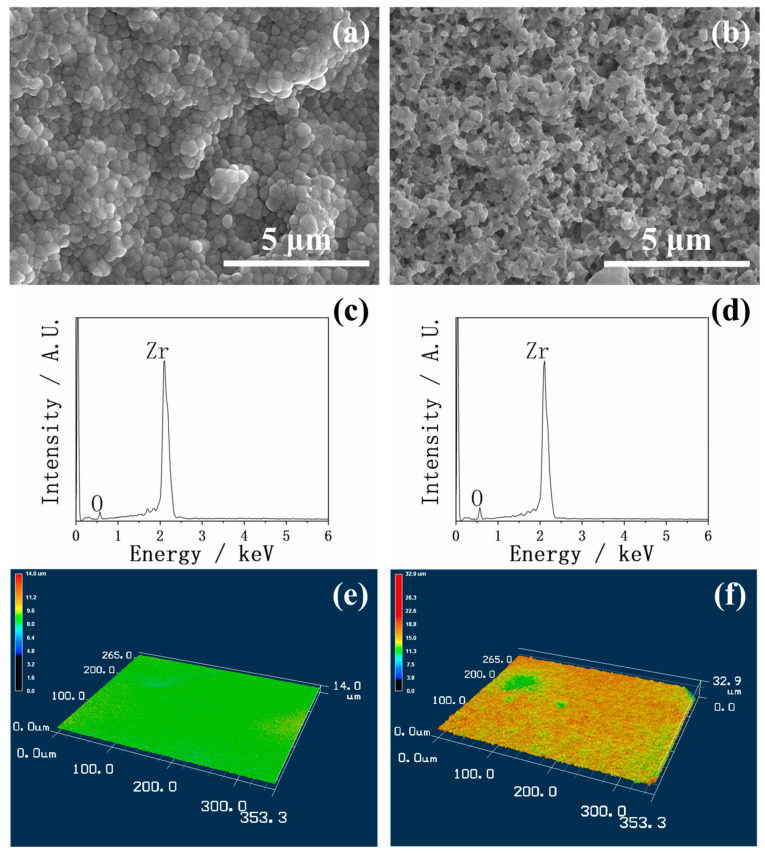
SEM (**a**,**b**), EDX (**c**,**d**), and 3D images (**e**,**f**) of YSZ plates before and after HF treatment. Before HF treatment: (**a**,**c**,**e**). After HF treatment: (**b**,**d**,**f**).

In Figure 1, the SEM, EDX, and 3D images of YSZ plates before and after HF treatment are shown. Figure 1a shows the YSZ surface morphology before HF treatment. The surface was densely packed with relatively uniform nanoparticles, forming a compact structure. After HF treatment, as shown in Figure 1b, a significantly rougher and more porous morphology was observed. The particles were smaller, more irregular, and less densely packed, indicating that the HF etching effectively increased the surface roughness and introduced more micropores. This morphological transformation could provide a larger surface area, which is beneficial for the precipitation of calcium phosphate.

Although significant morphological differences were observed in the SEM before and after HF treatment, the EDX in Figure 1c,d revealed no substantial difference in the elemental compositions between the two surfaces, indicating that the HF treatment primarily modified the surface topography rather than the chemical composition.

The 3D images further demonstrated the effect of the HF treatment on the YSZ plates in Figure 1e,f. Compared with the untreated surface, which appeared relatively flat and smooth, the HF-treated surface exhibited a much more irregular and roughened morphology, consistent with the morphological features observed in the SEM.

**Figure 2:**
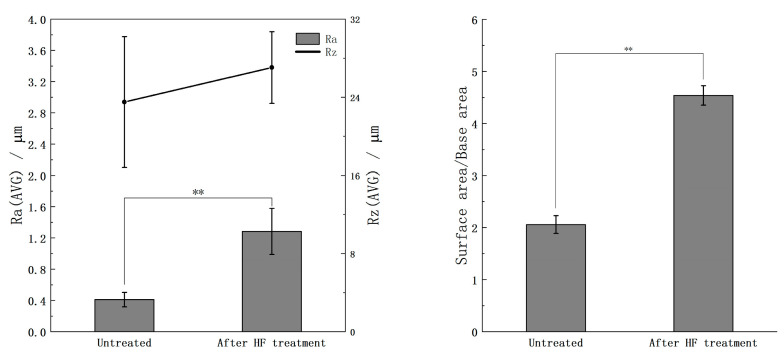
Ra, Rz, and surface area/base area of YSZ plates before and after HF treatment. Ra: average roughness; Rz: maximum roughness depth. The symbol “**” indicates *p* < 0.01, and the absence of a symbol indicates *p* > 0.05, as determined by Student’s *t*-test.

Figure 2 shows the quantitative roughness and surface area measurements, which also support these observations: the average roughness (Ra) increased from approximately 0.4 µm for the untreated surface to over 1.2 µm after the HF treatment, while the maximum roughness depth (Rz) also showed an increase. The ratio of surface area to base area significantly increased by more than twofold after HF etching. These results confirm that the HF treatment effectively enhanced the surface roughness of the YSZ substrates, thereby enlarging the effective contact area and potentially providing more favorable nucleation sites for the subsequent calcium phosphate precipitation.

**Figure 3:**
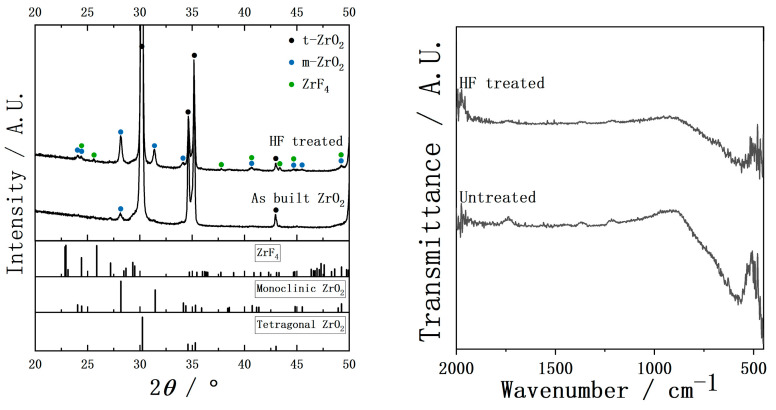
XRD and FTIR of YSZ plates before and after HF treatment.

The XRD and FTIR of the YSZ plates before and after HF treatment are shown in Figure 3. Before HF treatment, the XRD showed that the main diffraction peaks corresponded to tetragonal ZrO_2_, with only a few minor peaks assigned to monoclinic ZrO_2_, indicating that the sample predominantly consisted of the tetragonal phase with a small amount of the monoclinic phase. After HF treatment, the intensity of the peaks belonging to monoclinic ZrO_2_ increased, suggesting an increase in the relative proportion of the monoclinic phase. Additionally, weak peaks corresponding to ZrF_4_ appeared in the XRD, indicating that trace residues remained on the surface despite 30 min of ultrasonic cleaning. The FTIR before HF treatment exhibited a strong and broad absorption band centered around 562 cm^−1^, which is attributed to the Zr-O in ZrO_2_. After HF treatment, the intensity of this band was noticeably reduced, suggesting a decrease in the quantity of surface Zr-O species.

### 2.2. After m-SBF Treatment

**Figure 4:**
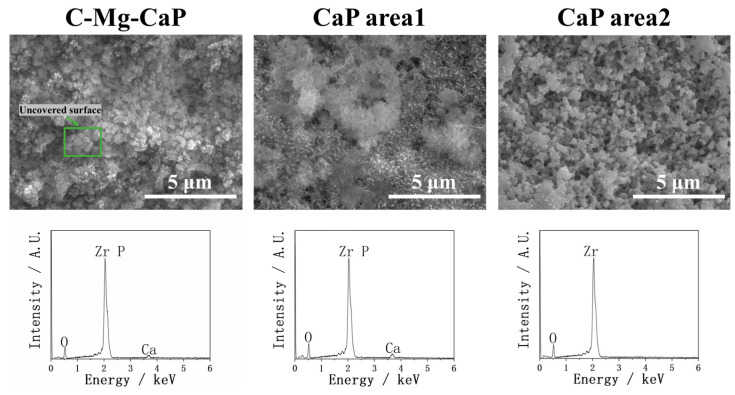
SEM and EDX of YSZ plates pretreated with C-Mg-CaP and CaP m-SBF. CaP area 1 and CaP area 2 represent different regions of the same sample.

In Figure 4, the coating performances of YSZ plates treated with C-Mg-CaP and CaP m-SBF are shown via SEM and EDX. The C-Mg-CaP m-SBF, which previously demonstrated an excellent coating ability on Zr-50Ti alloys [14], was applied to YSZ plates. However, it was found that on YSZ surfaces, C-Mg-CaP m-SBF failed to achieve complete surface coating and exhibited poor precipitation, as shown in the SEM, which significantly differed from its performance on Zr-50Ti alloys. Consequently, the C-Mg-CaP m-SBF was unsuitable for coating YSZ plates. In the EDX, since Zr and P overlapped, it was difficult to distinguish the intensity of P in calcium phosphate. However, from the figure, it is clear that the intensity of Ca is low. Subsequently, CaP m-SBF was applied to YSZ plates. As observed in our previous research [14], the CaP m-SBF promoted localized apatite precipitation in some areas of the substrate, while other areas remained completely uncovered, as shown in CaP area 1 and area 2 of Figure 4, resulting in severely non-uniform calcium phosphate coatings. And the EDX also proved the argument that there is a Ca peak in area 1 and absolutely no Ca peak in area 2. This non-uniformity indicates that the CaP m-SBF is also unsuitable for achieving uniform calcium phosphate layers on YSZ plates.

**Figure 5:**
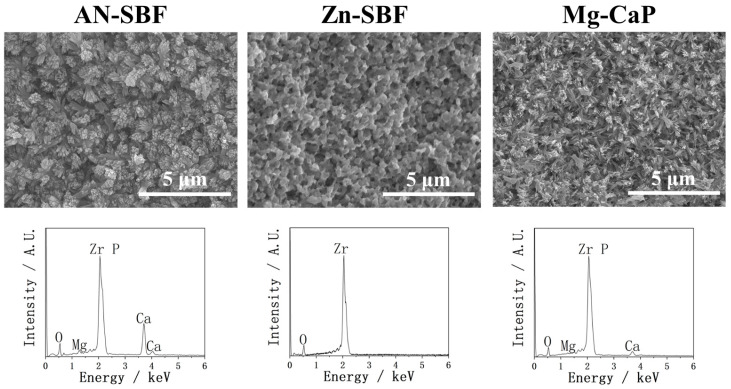
SEM and EDX of YSZ plates pretreated with AN-SBF, Zn-SBF, and Mg-CaP m-SBFs.

In Figure 5, the coating performances of YSZ plates treated with AN-SBF, Zn-SBF, and Mg-CaP m-SBFs are shown via SEM and EDX. The comparison between AN-SBF and Mg-CaP m-SBF revealed that, under the same coating conditions (pH 8.0 at 36.5 °C, 70 °C incubation for 1 day), group AN-SBF produced more substantial and denser calcium phosphate precipitates on YSZ plates compared to group Mg-CaP, according to SEM. In EDX, the Ca peak of group AN-SBF is much higher than that of group Mg-CaP, indicating a better coating performance. Considering the goal of incorporating Zn^2+^, Zn-containing m-SBFs were further explored. The introduction of Zn^2+^ into the SBF (Zn-SBF) completely inhibited the formation of calcium phosphate on YSZ plates; the uncovered YSZ surface was observed in SEM, and in EDX, the high-intensity Ca peak disappeared after the incorporation of Zn^2+^. These results show the total failure of the coating.

**Figure 6:**
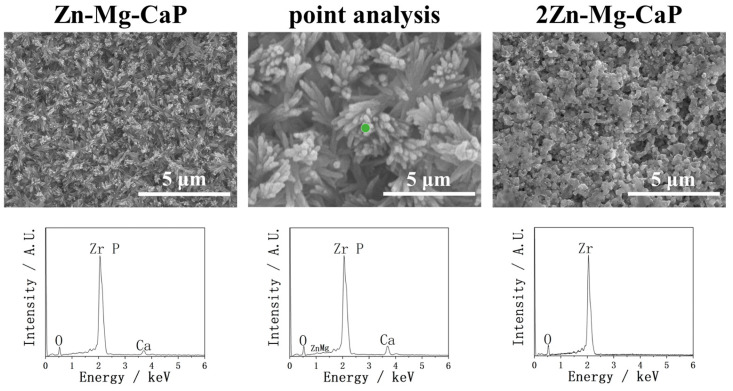
SEM and EDX of YSZ plates pretreated with Zn-Mg-CaP and 2Zn-Mg-CaP m-SBFs. The point analysis is a higher-magnification image of the same location in Zn-Mg-CaP. In “point analysis”, the green dot indicates the measurement location for the point analysis.

In Figure 6, the coating performances of YSZ plates treated with Zn-Mg-CaP and 2Zn-Mg-CaP m-SBF are shown via SEM and EDX. In contrast, the calcium phosphate was well precipitated in Zn-Mg-CaP m-SBF on YSZ plates. The surface morphology of the precipitates and the intensity of the Ca peak showed almost no change during this Zn-doping process between group Mg-CaP and group Zn-Mg-CaP. However, the Zn peak cannot be observed in EDX in group Zn-Mg-CaP at 10k magnification, and very weak Zn peaks could only be observed under point analysis at 50k magnification. Moreover, increasing the Zn^2+^ concentration (2Zn-Mg-CaP) again led to the complete suppression of apatite formation, suggesting that excessive Zn^2+^ has a strong inhibitory effect on calcium phosphate precipitation. Overall, Zn-Mg-CaP m-SBF provided the most favorable results for YSZ coating in this study, balancing effective apatite formation with the incorporation of Zn^2+^.

**Figure 7:**
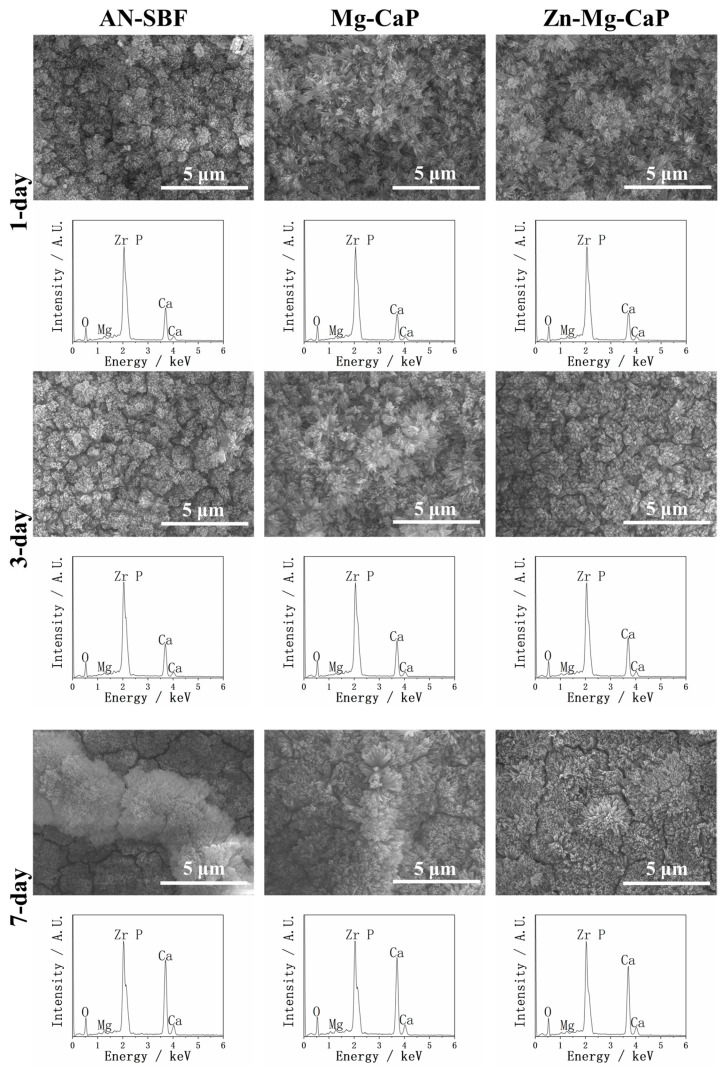
SEM and EDX of three types of m-SBF-pretreated YSZ plates soaked in standard SBF for 1, 3, and 7 days.

Considering that the CaP, C-Mg-CaP, Zn-SBF, and 2Zn-Mg-CaP m-SBFs were unsuitable for calcium phosphate precipitation on YSZ substrates, only AN-SBF, Mg-CaP, and Zn-Mg-CaP m-SBFs were evaluated for bioactivity. In Figure 7, the calcium phosphate-growing performances of three types of m-SBF-pretreated YSZ plates in standard SBF are shown via SEM and EDX. During the m-SBF pretreatment process, slight differences in the morphology of the calcium phosphate precipitates were observed among group AN-SBF, group Mg-CaP, and group Zn-Mg-CaP. These differences gradually diminished during the SBF soaking, as the calcium phosphate layers grew. Regardless of whether during the m-SBF pretreatment or the subsequent SBF soaking period, the surface morphologies of all three groups tended to exhibit rod-like structures. Furthermore, in the EDX, because the Zn peak overlaps with the Na peak at around 1 keV, and SBF contains a high concentration of NaCl, Zn detection under these conditions was inconclusive due to spectral interference.

Because all of the calcium phosphate formed in the three types of m-SBFs could further induce apatite formation after soaking in standard SBF, the three types of m-SBF-treated YSZ plates were confirmed to have bioactivity according to ISO 23317.

**Figure 8:**
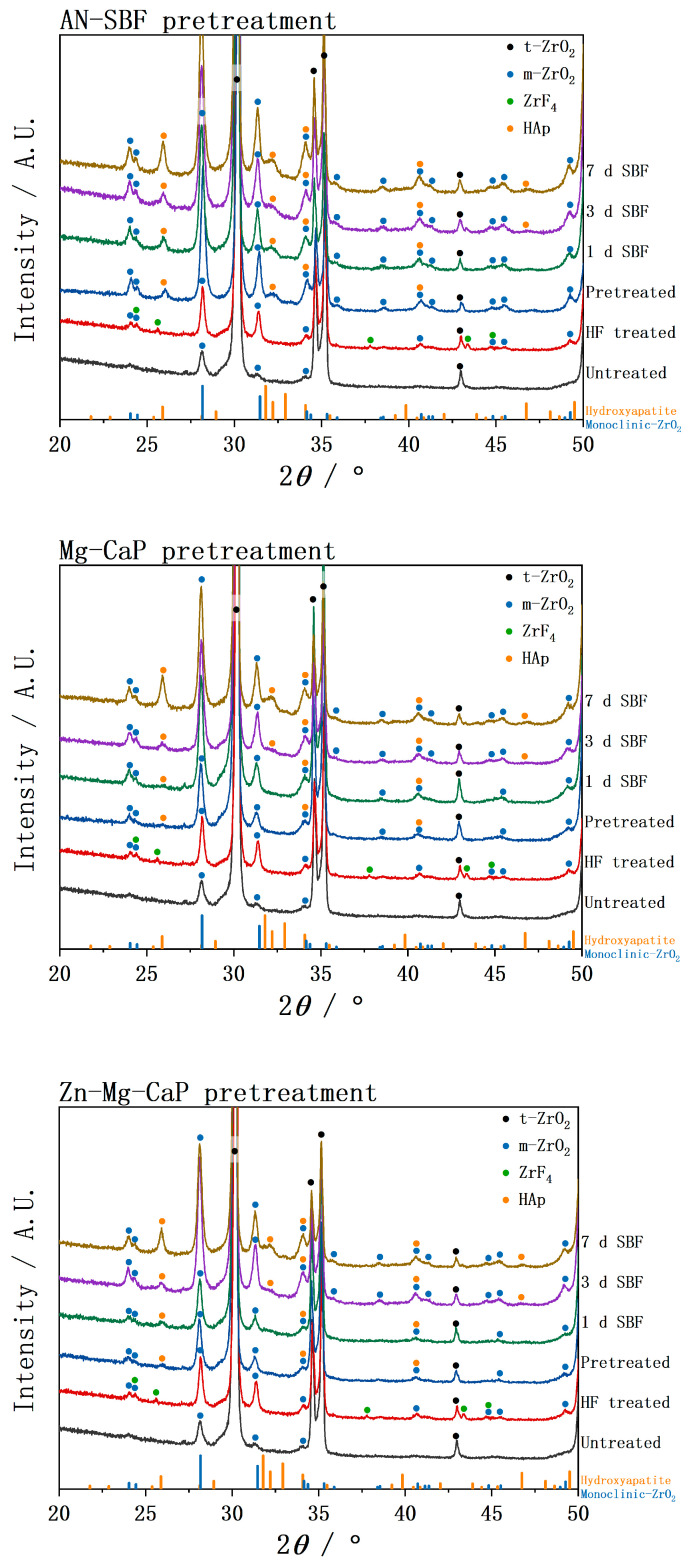
TF-XRD of untreated, HF-treated, three types of m-SBF-pretreated, and 1-, 3-, and 7-day SBF-soaked YSZ plates.

In Figure 8, the TF-XRD of the untreated, HF-treated, three types of m-SBF-pretreated, and SBF-soaked YSZ plate samples is shown. After m-SBF treatment, although the m-SBFs were different, the ZrF_4_ peaks disappeared, indicating the formation of a calcium phosphate surface layer that covered the YSZ. Among the three groups, the AN-SBF-treated samples showed notably enhanced monoclinic peak intensity immediately after treatment, whereas group Mg-CaP and group Zn-Mg-CaP exhibited only minor increases in monoclinic contents after m-SBF treatment but demonstrated progressive transition during the subsequent SBF soaking.

In all groups, hydroxyapatite characteristic peaks were observed after m-SBF treatment, suggesting that after 1 day of exposure to the high-temperature and high-pH calcium phosphate solution, not only had ANs formed as reported by Yao et al., but these ANs had also already initiated crystallization. Subsequently, these apatite peaks were further intensified after soaking for 1, 3, and 7 days in SBF. Notably, the main peak did not appear at the typical 31.8° corresponding to the (211) but at the 25.9° corresponding to the (002), indicating preferential growth along the c-axis. Furthermore, the sharpest and most intense apatite peaks were observed in group AN-SBF, whereas group Mg-CaP and group Zn-Mg-CaP showed less intense peaks, suggesting fewer precipitates or the lower crystallinity of the apatite.

**Figure 9:**
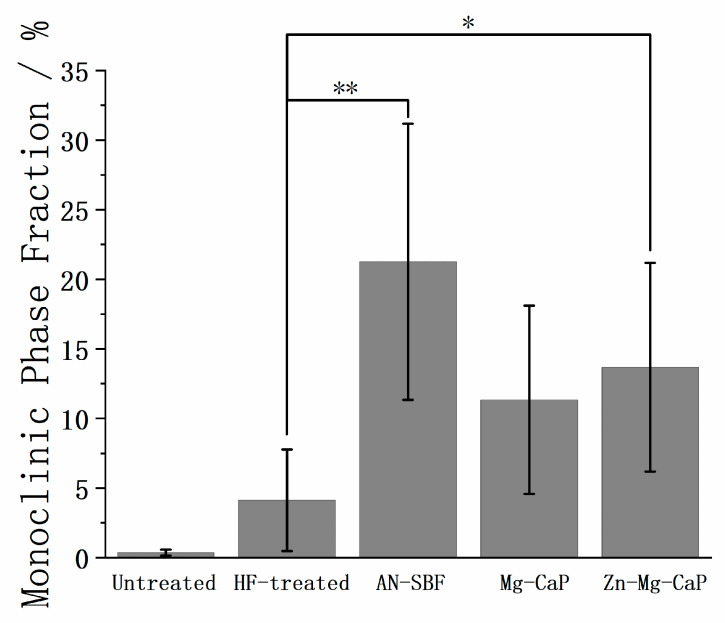
Monoclinic phase fractions of untreated, HF-treated, and three types of m-SBF-pretreated YSZ plates. The symbol “**” indicates *p* < 0.01, “*” indicates *p* < 0.05, and the absence of a symbol indicates *p* > 0.05, as determined by Student’s *t*-test.

In Figure 9, the monoclinic phase fractions of the untreated, HF-treated, and three types of m-SBF-pretreated YSZ plates are shown. The monoclinic phase fraction was calculated according to Toraya et al. [19], using the empirical calibration Formula (1), where *X_m_* (2) is the integrated intensity ratio of monoclinic ($\bar{1}11$) and (111) to tetragonal (101):

(1) $$V_{m}=\frac{1.311X_{m}}{1+0.311X_{m}}$$

(2) $$X_{m}=\frac{I_{m}(\bar{1}11)+I_{m}(111)}{I_{m}(\bar{1}11)+I_{m}(111)+I_{t}(101)}$$

A noticeable increase in the monoclinic phase fraction was detected after both HF and m-SBF treatments, indicating that the t→m transition occurred during these processes. The untreated YSZ exhibited only trace amounts of the monoclinic phase, whereas the HF treatment led to a measurable increase. After m-SBF soaking, all samples showed further t→m transition. Among the three types of m-SBFs, the AN-SBF treatment produced the highest monoclinic phase fraction, followed by Mg-CaP and Zn-Mg-CaP m-SBFs.

**Figure 10:**
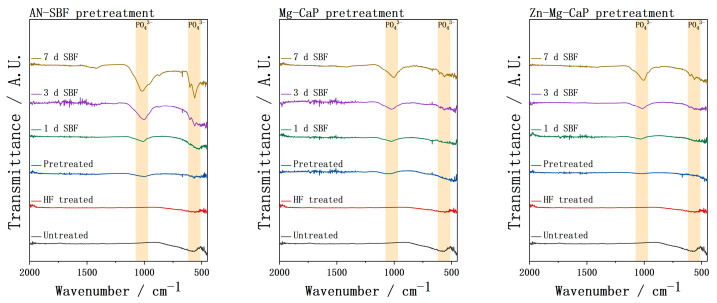
FTIR spectra of untreated, HF-treated, three types of m-SBF-pretreated, and 1-, 3-, and 7-day SBF-soaked YSZ plates.

In Figure 10, the FTIR spectra of the untreated, HF-treated, three types of m-SBF-pretreated, and SBF-soaked YSZ plate samples are shown. Compared with group Mg-CaP and group Zn-Mg-CaP, group AN-SBF showed stronger apatite formation from the pretreatment, as evidenced by the P-O bands. During the subsequent standard SBF soaking, these P-O bands increased more rapidly. In contrast, group Mg-CaP and group Zn-Mg-CaP showed comparable FTIR intensities over time; no obvious effect of Zn addition was observed within our detection limits.

**Figure 11:**
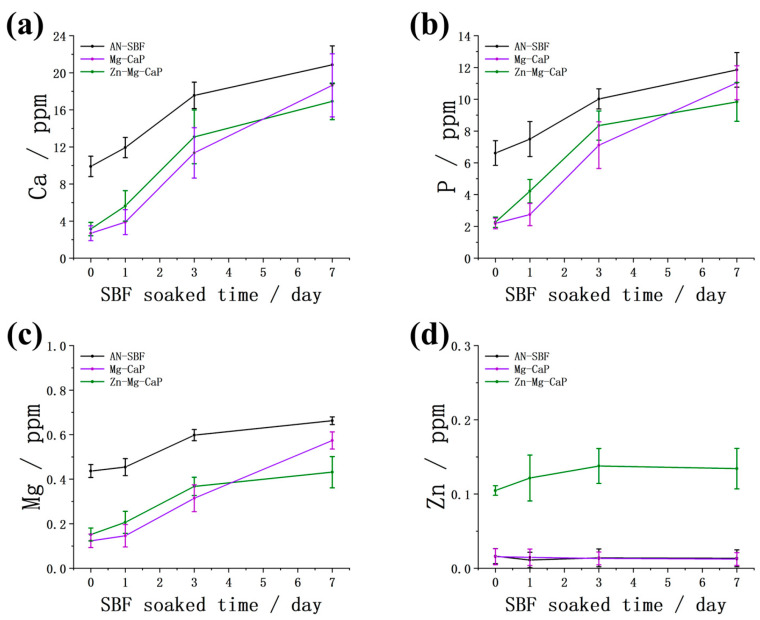
Concentrations of (**a**) calcium, (**b**) phosphorus, (**c**) magnesium, and (**d**) zinc in apatite formed on three types of m-SBF-pretreated YSZ plates after soaking in SBF for 0, 1, 3, and 7 days, as measured by ICP after dissolving samples in 50 mL of 1 M HNO_3_.

In Figure 11, the Ca, P, Mg, and Zn concentrations of the apatite coatings, which were dissolved in HNO_3_ solution, are shown. During the pretreatment stage, the Ca, P, and Mg concentrations in group AN-SBF were significantly higher than those of group Mg-CaP and group Zn-Mg-CaP, indicating that more apatite formed. With subsequent soaking in standard SBF, the Ca, P, and Mg concentrations in group Mg-CaP and group Zn-Mg-CaP gradually increased and approached the level of group AN-SBF by 7 days.

In addition, it should be noted that all samples underwent a 1-day immersion in ultrapure water after m-SBF pretreatment. This step caused the partial dissolution of the initially formed apatite, which explains why the Zn concentration at day 0 was relatively low in group Zn-Mg-CaP.

In Table 1, the Zn/Ca and Mg/Ca molar ratios of the apatite formed after m-SBF treatment are shown. The Zn/Ca ratio was approximately 3.33%, which was close to the Mg/Ca ratio, even though the Mg concentration (1.5 mM) in the m-SBF was 15 times higher than that of Zn (0.1 mM). This finding suggests that Zn ions exhibit a stronger affinity toward apatite than Mg ions. Furthermore, the similar Mg/Ca ratios among group AN-SBF, group Mg-CaP, and group Zn-Mg-CaP indicate that the addition of Zn did not significantly affect Mg incorporation, suggesting their possible co-precipitation within the apatite layer.

**Figure 12:**
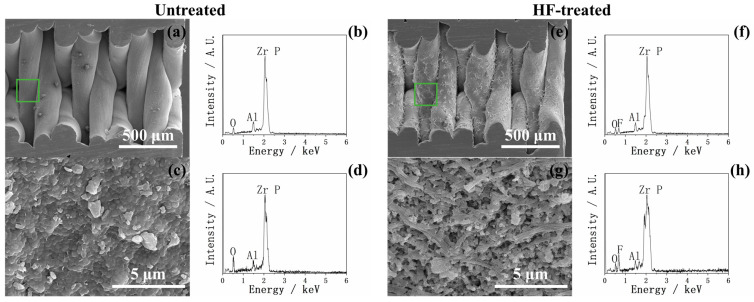
SEM and EDX images of the cross sections of untreated and HF-treated YSZ plates. (**a**) Low-magnification SEM image of the cross section of an untreated YSZ plate. (**b**) EDX corresponding to (**a**). (**c**) High-magnification SEM image of the green-marked area in (**a**). (**d**) EDX corresponding to (**c**). (**e**) Low-magnification SEM image of the cross section of an HF-treated YSZ plate. (**f**) EDX corresponding to (**e**). (**g**) High-magnification SEM image of the green-marked area in (**e**). (**h**) EDX corresponding to (**g**).

**Figure 13:**
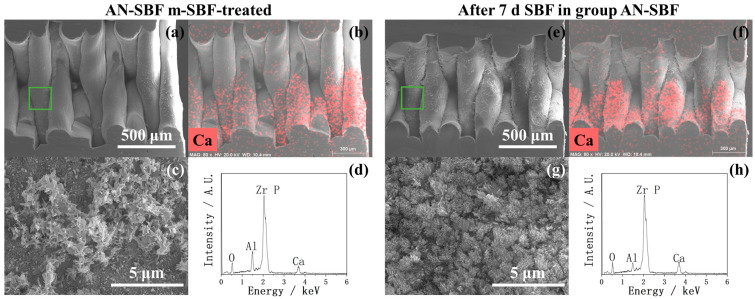
SEM and EDX of the cross sections of AN-SBF-pretreated and 7-day SBF-soaked YSZ plates. (**a**) Low-magnification SEM image of the cross section of an AN-SBF-pretreated YSZ plate. (**b**) EDX mapping corresponding to (**a**). (**c**) High-magnification SEM image of the green-marked area in (**a**). (**d**) EDX analysis corresponding to (**a**). (**e**) Low-magnification SEM image of the cross section of a 7-day SBF-soaked YSZ plate. (**f**) EDX mapping corresponding to (**e**). (**g**) High-magnification SEM image of the green-marked area in (**e**). (**h**) EDX analysis corresponding to (**e**).

**Figure 14:**
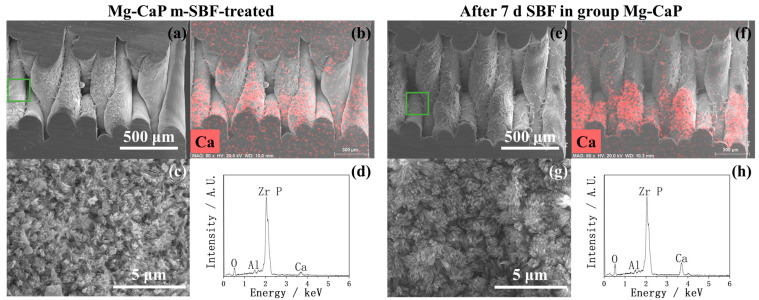
SEM and EDX of the cross sections of Mg-CaP-pretreated and 7-day SBF-soaked YSZ plates. (**a**) Low-magnification SEM image of the cross section of a Mg-CaP-pretreated YSZ plate. (**b**) EDX mapping corresponding to (**a**). (**c**) High-magnification SEM image of the green-marked area in (**a**). (**d**) EDX analysis corresponding to (**a**). (**e**) Low-magnification SEM image of the cross section of a 7-day SBF-soaked YSZ plate. (**f**) EDX mapping corresponding to (**e**). (**g**) High-magnification SEM image of the green-marked area in (**e**). (**h**) EDX analysis corresponding to (**e**).

**Figure 15:**
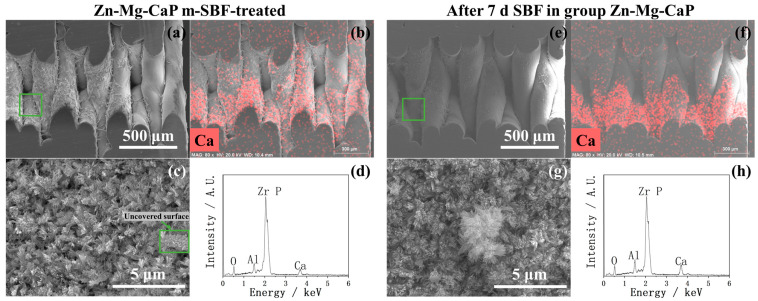
SEM and EDX of the cross sections of Zn-Mg-CaP-pretreated and 7-day SBF-soaked YSZ plates. (**a**) Low-magnification SEM image of the cross section of a Zn-Mg-CaP-pretreated YSZ plate. (**b**) EDX mapping corresponding to (**a**). (**c**) High-magnification SEM image of the green-marked area in (**a**). (**d**) EDX analysis corresponding to (**a**). (**e**) Low-magnification SEM image of the cross section of a 7-day SBF-soaked YSZ plate. (**f**) EDX mapping corresponding to (**e**). (**g**) High-magnification SEM image of the green-marked area in (**e**). (**h**) EDX analysis corresponding to (**e**).

In Figure 12, Figure 13, Figure 14 and Figure 15, the cross-sectional SEM images of the fused deposition modeling-printed porous YSZ are shown. Numerous fibrous residues are observed on the inner surfaces of the pores at low magnification in most samples. A strong Al peak was consistently detected in EDX. These residues are therefore considered to come from the remaining Al-containing additives or binders used during the 3D-printing process, which could not be completely removed from the inner pores.

In the EDX mappings shown in Figure 13, Figure 14 and Figure 15, the Ca appears more intense in the lower part of the image compared with the upper part. This uneven intensity mainly results from the 3D pore geometry and the surface inclination, which affect the X-ray detection efficiency, rather than from a real difference in Ca. However, as shown in Figure 13b, the Ca mapping shows a clear difference between the left and right sides of the image; that is, the Ca signal is higher on the outer surface of the pores and much weaker inside. In contrast, other mappings show nearly uniform color intensity, indicating that the uneven apatite deposition between the inner and outer pore appeared only in group AN-SBF during the m-SBF pretreatment and disappeared after 7 days of SBF soaking.

In Figure 13c, the high magnification of the cross-sectional SEM of group AN-SBF shows obviously less precipitation inside the pores compared with the outer surface, as shown in Figure 5. By comparing Figure 14c and Figure 15c, group Zn-Mg-CaP exhibits fewer precipitates compared with group Mg-CaP, as the uncovered surface of YSZ was observed. This difference was not observed on the outer surface, as shown in Figure 5 and Figure 6.

**Figure 16:**
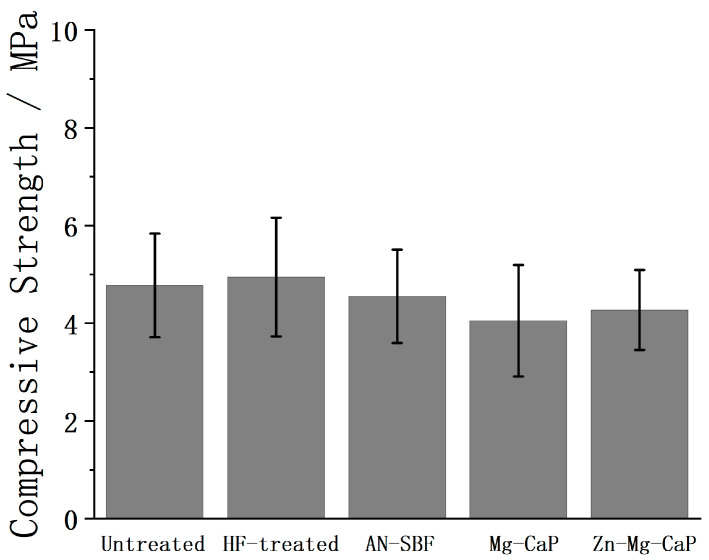
Compressive strengths of untreated, HF-treated, and three types of m-SBF–pretreated YSZ plates after subsequent 7-day SBF soaking.

In Figure 16, the compressive strengths of the untreated, HF-treated, and three types of m-SBF–pretreated YSZ plates after 7-day SBF soaking are shown. After 7 days of SBF immersion, a dense apatite layer was observed on the HF-pretreated porous YSZ substrates, accompanied by partial t→m transition detected by XRD. However, the compressive strengths of the samples showed no statistically significant difference compared to the as-prepared substrates, indicating that the apatite deposition did not enhance the mechanical properties and the t→m transition did not impair them.

## 3. Discussion

In Figure 8 and Figure 9, the XRD and phase fraction reveal a clear t→m phase transition on the surfaces of the HF-treated YSZ plates. This observation is consistent with the findings of Flamant et al. [20], who demonstrated that the HF etching of 3Y-TZP increased the monoclinic phase content at the surface regardless of the etching duration. Besides HF treatment, water itself can also induce the t→m transition of ZrO_2_. Xie et al. [21] reported that exposure of tetragonal ZrO_2_ to water vapor or liquid water at room temperature induces the transition to the monoclinic phase without any significant surface area loss. In Figure 8 and Figure 9, the t→m transition is observed in all m-SBF treatments. Since no previous reports have indicated that calcium phosphate itself causes such a phase transition, this transition is likely attributable to the aqueous environment of the m-SBFs. Interestingly, in our experiments, a more pronounced t→m transition was observed in YSZ soaked in high-temperature and high-pH SBF (AN-SBF) compared with the same conditions in CaP-containing solutions (Mg-CaP and Zn-Mg-CaP). To the best of our knowledge, this specific phenomenon has not been reported before. Although the underlying mechanisms remain unclear, this finding suggests that the differences between SBF and CaP solutions may significantly affect the low-temperature degradation behavior of YSZ, warranting further systematic investigation.

Sato et al. [22] investigated the influence of water on the t→m phase transition in YSZ and proposed that water molecules react with Zr-O-Zr bonds in the tetragonal lattice, breaking these linkages and forming surface Zr-OH groups. Uchida et al. [23] later reported that such surface Zr-OH, formed on Ce-TZP/Al_2_O_3_, plays a crucial role in inducing the nucleation of bonelike apatite in SBF. Barrère et al. [24] reported that increasing the NaCl concentration delayed homogeneous precipitation in the solution and thereby altered the Ca-P deposition pathway. However, in our experiments, a more pronounced t→m transition occurred in group AN-SBF, which likely facilitated the generation of additional Zr-OH groups. These hydroxylated sites could serve as potent nucleation centers for hydroxyapatite, offsetting the delay effects of NaCl. This mechanism may explain the unexpectedly accelerated apatite precipitation observed in the NaCl-containing SBF compared with NaCl-free CaP solutions.

In group AN-SBF, the outer surfaces of the pores exhibited much faster apatite precipitation than the inner pores, as shown in Figure 13b. This trend is opposite to the commonly observed phenomenon, where apatite nucleation and growth preferentially occur inside macropores rather than on flat surfaces, as reported by Bianchi et al. [25]. This difference may be attributed to the t→m phase transition occurring at the outer surface. As described in the previous section, the t→m transition facilitates apatite nucleation, thereby significantly accelerating calcium phosphate precipitation on the outer surface. Consequently, precipitation inside the pores was slower than that on the outer surface.

In group Mg-CaP and group Zn-Mg-CaP, the apatite precipitation appeared more uniform between the inner and outer pores compared with group AN-SBF. This may be because the t→m transition-induced enhancement at the outer surface and the geometrical promotion inside the pores were comparable, leading to a similar overall degree of apatite formation both inside and outside the pores.

In Figure 8, the XRD revealed that the apatite formed in all three m-SBF groups was dominated by the (002), indicating a strong c-axis-preferred orientation. This observation is in line with the findings of Müller et al. [26], who reported that apatite crystals formed in biomimetic supersaturated solutions preferentially grow along the c-axis and exhibit a pronounced [002] orientation, closely resembling the structural orientation of biological apatite. Consistently, the SEM images in Figure 4, Figure 5, Figure 6 and Figure 7 show vertically aligned, rod-like apatite structures, further supporting the presence of c-axis-oriented crystal growth. Such a morphology is comparable to that in the report by Pang et al. [27], who demonstrated that hydroxyapatite coatings with a (002) plane formed vertically aligned, rod-like nanoarrays and exhibited superior surface properties, including enhanced hydrophilicity, fibronectin adsorption, and osteogenic differentiation.

Ding et al. [12] reported that Mg^2+^ strongly inhibits the crystallization of hydroxyapatite, leading to a pronounced reduction in the hydroxyapatite peak intensity in XRD. This observation is consistent with our results, where SEM, EDX, and FTIR confirmed significant apatite deposition on the substrate surface, while the corresponding XRD displayed only weak apatite peaks. These findings suggest that the incorporation of Mg^2+^ into the CaP phase suppressed the crystallinity of apatite, thereby accounting for the low diffraction intensity in group Mg-CaP in our study. Ortiz et al. [16] demonstrated that Zn^2+^ also plays an inhibitory role in apatite crystallization. In this study, however, such inhibitory effects were not clearly observed. Based on the surface SEM observations, as well as the subsequent XRD, FTIR, and ICP analyses, as shown in Figure 4, Figure 5, Figure 6, Figure 7, Figure 8, Figure 9, Figure 10 and Figure 11, no significant differences were found between group Mg-CaP and group Zn-Mg-CaP. The only noticeable difference appeared in the cross-sectional SEM images, as shown in Figure 14 and Figure 15, at the center regions of the YSZ pores, where group Mg-CaP still exhibits good apatite coverage on the pore surfaces, whereas in group Zn-Mg-CaP, some uncovered YSZ areas are observed, indicating a decreased apatite ingrowth ability in porous YSZ.

## 4. Materials and Methods

### 4.1. Materials

The fused deposition modeling-3D-printed porous 5.4 mol% YSZ (Hit Research, Kyoto, Japan) was fabricated at a size of 10 × 10 × 2.5 mm^3^ with a pore diameter of 0.6 mm and a porosity of approximately 16%.

### 4.2. Hydrofluoric Acid Treatment

The as-built YSZ plates were soaked in 55 wt% HF (Merck Ltd./Sigma-Aldrich Japan G.K., Tokyo, Japan) for 3 h. After HF soaking, the YSZ plates were treated by ultrasonic cleaning in distilled water for 30 min and air-dried for 1 day.

### 4.3. Preparation of Standard SBF and m-SBFs

SBF has been reported to not only simulate the bioactivity of biomaterials in vitro [8] but to also fabricate nanoparticles termed ANs [9]. In this study, we prepared a standard SBF (pH 7.4, 36.5 °C) according to ISO 23317 and m-SBFs (pH 8.0, 36.5 °C). The standard SBF was used to evaluate the bioactivity, while the m-SBFs were used to induce the precipitation of ANs, precursors of apatite.

Standard SBF was prepared by dissolving reagent-grade NaCl (FUJIFILM Wako Pure Chemicals, Osaka, Japan, 99.5%), NaHCO_3_ (Hayashi Pure Chemicals, Osaka, Japan, 99.5%~), KCl (Hayashi Pure Chemicals, Osaka, Japan, 99.5%), K_2_HPO_4_·3H_2_O (Nacalai Tesque, Kyoto, Japan, 99.0%), MgCl_2_·6H_2_O (Hayashi Pure Chemicals, Osaka, Japan, 98.0%), CaCl_2_ (FUJIFILM Wako Pure Chemicals, Osaka, Japan, 95.0%), and Na_2_SO_4_ (Hayashi Pure Chemicals, Osaka, Japan, 99.0%) in ultrapure water. The pH was adjusted to 7.40 with tris(hydroxymethyl)aminomethane ((CH2OH)_3_CNH_2_, FUJIFILM Wako Pure Chemicals, Osaka, Japan, 95.0%) and 1 M HCl (FUJIFILM Wako Pure Chemicals, Osaka, Japan) at 36.5 °C.

The m-SBFs were developed by modifying the ion concentrations of standard SBF and introducing Zn^2+^ through the dissolution of Zn(NO_3_)_2_·6H_2_O (FUJIFILM Wako Pure Chemicals, Osaka, Japan, 99.0%). Then, the pH of the m-SBFs was adjusted to 8.0 by dissolving (CH_2_OH)_3_CNH_2_ at 36.5 °C for supersaturating calcium phosphate. The ion concentrations of human blood plasma, SBF, and various m-SBFs are shown in Table 2.

### 4.4. m-SBF Pretreatment

The HF-treated YSZ plates were soaked in various m-SBFs at 70 °C for 1 day to accelerate the precipitation of calcium phosphate. After this process, the YSZ plates were coated with calcium phosphate layers depending on the immersion solution. Samples treated only with m-SBFs were soaked in ultrapure water for 1 day after the reaction, as AN-SBF contains NaCl. The m-SBF-treated and then SBF soaking samples were briefly rinsed with ultrapure water between the two steps.

### 4.5. Evaluation of Bioactivity

The apatite-forming ability, which was defined as a property to evaluate the bioactivity of biomaterials, was evaluated by soaking the m-SBF-treated samples in SBF for 1, 3, and 7 days at 36.5 °C. After SBF soaking, the SBF-soaked samples were also rinsed in ultrapure water for 1 day to remove NaCl.

### 4.6. Surface Characterization

Characterization of the YSZ plates was performed using several techniques. Thin-film X-ray diffraction (XRD) (X’Pert PRO, PANalytical, Almelo, Netherlands) was carried out with a CuKα radiation source operated at 45 kV and 40 mA. The surface morphologies were examined by scanning electron microscopy (SEM) (SU6600, Hitachi High-Tech, Tokyo, Japan) coupled with energy dispersive X-ray spectroscopy (EDX) (XFlash 5010, Bruker, Billerica, MA, USA). Chemical bonding information was obtained using Fourier-transform infrared (FTIR) spectroscopy equipped with a diamond ATR method (FT/IR-4700, JASCO, Tokyo, Japan) under a resolution of 2 cm^−1^ and 100 scans.

### 4.7. Measurement of the Element Composition of Apatite by Inductively Coupled Plasma Atomic Emission Spectroscopy (ICP)

The m-SBF-treated or SBF-soaked YSZ plates were immersed in 50 mL of 1 M HNO_3_ (FUJIFILM Wako Pure Chemicals, Osaka, Japan) and kept sealed at room temperature for 3 days to completely dissolve the apatite layer. The elemental concentrations of Ca, P, Mg, and Zn were determined using inductively coupled plasma atomic emission spectroscopy (ICPS-7510, Shimadzu, Kyoto, Japan). Calibration curves were obtained from standard stock solutions (1000 mg/L each) (FUJIFILM Wako Pure Chemicals, Osaka, Japan) of the respective elements, which were diluted with ultrapure water.

### 4.8. Cross-Sectional Characterization

The samples were sectioned from the middle to expose the cross section of the porous structure, and the SEM and EDX were carried out.

### 4.9. Compressive Strength

The compressive strengths of the apatite-coated YSZ substrates soaked in SBF for 7 days were measured according to the modified ASTM C1424 method. YSZ plates were placed between two parallel platens of a universal testing machine (Autograph AGS-H, Shimadzu, Kyoto, Japan). A compressive load was applied at a crosshead speed of 0.5 mm/min until failure occurred.

## 5. Conclusions

The 3D-printed porous YSZ substrates etched with HF exhibited a roughened surface and induced a partial t→m phase transition. During the m-SBF treatment, Mg^2+^ ions refined the apatite microstructure and improved the coating uniformity, while excessive Zn^2+^ suppressed calcium phosphate precipitation. Compared with standard SBF under high-temperature and high-pH conditions, the calcium phosphate m-SBF solution caused much less t→m phase transition on YSZ substrates. The apatite layer formed in Mg^2+^- and Zn^2+^-containing m-SBF achieved an optimal balance, exhibiting uniform precipitation and causing reduced t→m phase transition. The compressive strength of the YSZ substrates remained unchanged after apatite formation, indicating that the treatment did not compromise mechanical strength. Through these processes, the obtained YSZ exhibited improved surface bioactivity and the potential for application as an osteoconductive ceramic implant material.

## Figures and Tables

**Table 1:** Zn/Ca and Mg/Ca molar ratios (%) of apatite formed on three types of m-SBF-pretreated YSZ plates.

	AN-SBF	Mg-CaP	Zn-Mg-CaP
Zn/Ca	-	-	3.33
Mg/Ca	4.41	4.59	4.82

**Table 2:** Ion concentrations of human blood plasma, SBF, and seven types of m-SBFs.

Ion Concentration (mM)									
	Blood Plasma	SBF	AN-SBF	Zn-SBF	CaP	Mg-CaP	Zn-Mg-CaP	2Zn-Mg-CaP	C-Mg-CaP
Na^+^	142.0	142.0	142.0	142.0	-	-	-	-	4.2
K^+^	5.0	5.0	5.0	5.0	2.0	2.0	2.0	2.0	2.0
Mg^2+^	1.5	1.5	1.5	1.5	-	1.5	1.5	1.5	1.5
Ca^2+^	2.5	2.5	2.5	2.5	2.5	2.5	2.5	2.5	2.5
Cl^−^	103.0	147.8	147.8	147.8	5.0	8.0	8.0	8.0	8.0
HCO_3_^−^	27.0	4.2	4.2	4.2	-	-	-	-	12.5
HPO_4_^2−^	1.0	1.0	1.0	1.0	1.0	1.0	1.0	1.0	1.0
SO_4_^2−^	0.5	0.5	0.5	0.5	-	-	-	-	-
Zn^2+^	0.012–0.018	-	-	0.1	-	-	0.1	0.2	-
pH (36.5 °C)	7.2–7.4	7.4	8.0	8.0	8.0	8.0	8.0	8.0	8.0

## Data Availability

Data are contained within the article.
